# University students’ behavioral intention and gender differences toward the acceptance of shifting regular field training courses to e-training courses

**DOI:** 10.1007/s10639-021-10701-1

**Published:** 2021-09-14

**Authors:** Abdullah M. Alghamdi, Dhaifallah S. Alsuhaymi, Fahad A. Alghamdi, Ahmed Mohamed Farhan, Saleh M. Shehata, Mona Mostafa Sakoury

**Affiliations:** 1 Department of Management Information Systems, College of Applied Studies and Community Service, Imam Abdulrahman Bin Faisal University, Dammam, 31441 Saudi Arabia; 2 Department of Curriculum and Instruction, College of Education, Imam Abdulrahman Bin Faisal University, Dammam, 31441 Saudi Arabia; 3 Department of General Courses, College of Applied Studies and Community Service, Imam Abdulrahman Bin Faisal University, Dammam, 31441 Saudi Arabia; 4 Department of Financial Management, College of Applied Studies and Community Service, Imam Abdulrahman Bin Faisal University, Dammam, 31441 Saudi Arabia

**Keywords:** E-training, E-Learning, Distance, learning, Acceptance model, Field Training Course

## Abstract

During the COVID-19 lockdown, all the courses at Imam Abdulrahman Bin Faisal University (IAU) were delivered fully online, including field-training courses. Since there was no previous experience in offering field-training courses in a distance format, the current study aims to identify factors that could impact students’ behavioral intention to accept the e-training approach in teaching field training courses at IAU. In order to gather the data, the researchers designed a questionnaire based on the UTAUT model and they ensured the face, content, and construct validity of the questionnaire by sending it to five experts in the relevant field and by using exploratory factor analysis. Also, all the questionnaire’s items were reliable since the Cronbach’s alpha values were above 0.77 for all the items. A total of 397 participants provided valid responses. The result of this study indicated that Effort Expectancy (EE), Facilitating Condition (FC), Performance Expectancy (PE), and Social Influence (SI), respectively were the primary predictors for students’ intention to use e-training. These factors explained 32.1% of the variance in students’ behavioral intentions. As far as students’ gender is concerned, there were significant differences between students’ PE, FC, and SI. Based on these results, policymakers at IAU will have a clear image of the most essential factors that colleges should target to increase students’ acceptance of e-training.

## Introduction

In the twenty-first century, Information and Communication Technology (ICT) is widely used in universities. It has become not only a valuable means to support learning via e-learning (Mahande & Malago, 2019), but also an appropriate new form of training delivery via e-training or remote training (Sarabadani et al., 2017). The term e-training expresses the use of technology in training, whether the training takes place face-to-face, through a medium, or entirely online. E-training also expresses the facilitation and technical support of training knowledge, in order to enhance performance at work and achieve personal or organizational goals (Zainab, Awais Bhatti, &Alshagawi, 2017).

E-training is considered a significant and sufficient alternative to traditional face-to-face training, particularly in unexpected crises such as the COVID-19 pandemic that precluded completion of this training course. Therefore, this year, students were only allowed to take the course as an online training skills course. Consequently, it is essential to examine students’ perceptions about the use of this technology (Fallery et al., 2010) for the field training course as a distance learning course.

Besides the ability of e-training to continue the training courses in such circumstances, e-training also has some features that overcome possible challenges that might accompany face-to-face training, such as the long distances between the trainees and their training workplaces, insufficient time assigned for meetings between trainers and trainees, especially when the trainer supervises many trainees in different locations, as well as logistics, transportation costs, and the possible difficulty in accessing training materials and requirements (Loh et al., 2013; Zainab et al., 2017).

The Field Training course is one of the compulsory courses that most students at Imam Abdulrahman Bin Faisal University (IAU) must take. This course helps students to experience the work off-campus in a job related to their field of study in real circumstances before they graduated. Moreover, it is worth mentioning that this course cannot be delayed due to the large number of students who have to register on it each semester, especially when there are limited opportunities for training in the labor market as there are other universities and institutes in the regain who seek the same opportunity for their students each semester. Based on the previous reasons along with the lockdown as a result of COVID-19, which made applying for the regular field-training course impossible, the decision-makers at IAU decided to shift the field training course from face-to-face training to an e-training course. Therefore, the curriculum developers redesigned the whole course to make it appropriate for delivering in an online format. The course was uploaded on the Blackboard and the course’s pages and sections were based on the Quality Matters standards.

The course took place during the Summer Semester of 2019–2020 which lasted for eight weeks. The course was divided into two main parts. The first part lasted for three weeks and provided a general labor overview and labor and management skills; whilst the remaining weeks were for the second part, which concentrated on linking students’ major skills and knowledge with labor market needs. Different teaching and learning strategies were used to benefit the course. Every week there were three synchronous and asynchronous lectures. After each lecture, students had to participate in different learning activities that matched the weekly topics, such as role-playing for workplace scenarios and case studies, and problem-solving using breakdown rooms. Blackboard and its features such as discussion forums, quizzes, polls, wiki, and communication tools were used in the course. Also, Zoom software was used for webinars and group meetings. In addition, there were weekly quest speakers from the labor market who talked and shared experiences with the students regarding the weekly topics and answered students’ questions and concerns about the real work environment.

This research aims to investigate factors that influence students’ behavior intention toward taking the field training course as a distance-learning course using the key factors of the Unified Theory of Acceptance and Use Technology (UTAUT), which are EE, PE, SI, and FC. However, the moderate constructs (e.g., gender, age) of the UTAUT model will be eliminated, as they are not within the investigation aim of the current study.

Conducting this research is considered necessary for policymakers at IAU and related colleges and universities. It helps in evaluating the experience of converting the traditional field training course into an electronic course. It also helps in developing a clear policy for implementing e-training and improving it continuously; in addition to investigating how well the students received the e-training course.

Further, no study so far has examined the effect of students’ acceptance of E-training on universities in Saudi Arabia, which shows the need to evaluate E-training acceptance to identify the contributing factors that could affect students’ acceptance of E-training. Thus, this research will enrich the literature in the E-training field.

## Theoretical framework

Individual adoption of technology is one of ICT research’s richest streams, with several models explaining the Behavioral Intention (BI) to adopt an innovative technology (Sarabadani et al., 2017). After reviewing prior studies, several studies were adopted on the Technology Acceptance Model (TAM) to detect factors that might impact students’ use of technology tools for learning purposes; the majority of these studies adopted UTAUT model to identify primary factors that impact students’ adoption of e-learning, whilst only a few studies focused on E-training. Examples of those studies are as follows:

Using a modified version of TAM as the theoretical framework, Vululleh (2018) examined factors that might impact post-secondary students’ adoption of technology for learning purposes in country of Liberia. Two new dimensions, namely, social influence (SI) and quality of life (QL), were added to the TAM constructs. Data were gathered from 269 students. The results revealed that a student’s intention to adopt e-learning is significantly impacted by the student’s perceived usefulness of e-learning, the student’s perceived usability of e-learning, and social influence.

Tarhini et al. (2015) also used a modified version of TAM in order to examine the elements that make technology accepted by students in higher education in a multicultural context. Data were collected from 1,173 students from two private universities, the first a Lebanese university and the second a British university. The results of this study refer to perceived ease of use, usefulness, social influence, self-efficacy, quality of work life, and facilitation conditions as primary factors in influencing students’ intention towards using e-learning. Overall, the aforementioned factors explain 69% of the change in behavioral intention for the sample from the British university and 57% for the sample from the Lebanese university.

Using the UTAUT model, Tan (2013) conducted a study to investigate the elements that cause university students to use e-learning sites in Taiwan. Data for this study were collected from 176 students from more than ten faculties. The results show that there is a positive effect of performance expectations, effort expectancy, and social influence on the intentions of Taiwanese students to use e-learning websites. Facilitating conditions and students’ intentions positively affect college students’ usage of e-learning websites. Further, the study supports the validity of UTAUT in investigating the intention of students to use e-learning sites.

Similarly, Escobar-Rodríguez et al. (2014) used modified version of the UTAUT model to investigate students’ intentions to adopt Facebook for learning. In this extended UTAUT version, the behavioral intention construct was mediated only by two added constructs, which were the perceived advantages of Facebook for learning and the perceived relevance of Facebook as a social media tool. This study included 956 students at a Spanish university. The results show that the perceived advantages of Facebook for students and the relevance of Facebook as a social media tool positively affect students’ intention to use Facebook as a learning tool. Also, performance expectancy and effort expectancy have a significant correlation with the perceived advantages of Facebook for students. Further, social influence and facilitating conditions have a significant relationship with the predictors of the perceived advantages of Facebook for students. Finally, the results concluded that UTAUT is a good model fit.

In the same context of e-learning, Haris and Sugito (2015), used a modified version of the UTAUT model to identify factors that impact student’s intention and use of ClassCraft e-learning. ClassCraft is a website that employs gamification principles for learning. This study included 83 students majoring in Computer Science and Information Systems at Tarumanagara University in Indonesia. The results revealed that course quality and social influence did not influence students’ intention to use ClassCraft. On the other hand, student motivation toward e-learning and student intention did significantly predict students’ adoption of ClassCraft.

Further, Ngampornchai and Adams (2016) conducted a study on 84 undergraduates to identify college students’ acceptance of using e-learning in Thailand using UTAUT. The study outcome showed that performance expectancy and effort expectancy, and social influence positively influenced students’ acceptance of e-learning. Also, the result revealed that there was a strong relationship between students’ intention to adopt e-learning and students’ class levels. Senior students were more inclined to accept e-learning than freshmen and junior students.

conducted a study on 84 undergraduates to identify college students’ acceptance of using e-learning in Thailand using UTAUT. The study outcome showed that performance expectancy, effort expectancy, and social influence positively influenced students’ acceptance of e-learning. Also, the result revealed that there was a strong relationship between students’ intention to adopt e-learning and students’ class levels. Senior students were more inclined to accept e-learning than freshmen and junior students.

Moreover, Mahande and Malago (2019) investigated factors that could predict graduate students’ usage of e-learning at Universitas Negeri Makassar in Indonesia by using the UTAUT model. This study included 170 students. Results indicate that facilitating conditions were the most influencing factors on student intention, followed by social influence, performance expectancy, and effort expectancy. Students’ intention and facilitating conditions were significantly able to predict graduate students’ use of e-learning.

Likewise, in the Saudi context, Alghamdi (2017) investigated factors that could affect university students’ acceptance of mobile learning as an e-learning tool. The researcher also examined the differences between participants according to their genders and specialty. His study included 1,541 students, and 451 instructors from three Saudi Universities. The results regarding students showed that self-efficacy, perceived usefulness, social influence, habits, and hedonic motivation factors directly influenced BI. Moreover, Alghamdi also found that the female students had higher mean scores than male students on most of the main factors. Another finding indicated that there were significant differences between male and female participants regarding three factors, which were PU, AT, and BI.

In the context of E-training, Zainab et al. (2017) tested perceived cost, perceived ease of use, and perceived usefulness as predictors for adoption of E-training in the Nigerian civil service. This study included 450 heads of departments. Perceived cost and perceived usefulness were significant predictors of E-training adoption. However, perceived ease of use did not have a significant relationship with E-training adoption.

Fallery et al. (2010) also conducted another study in the context of E-training acceptance. The researchers investigated factors that could influence employees to accept the use of videoconferencing to deliver training. The sample for this study was 60 employees in a French company. The results indicated that perceived usefulness was a significant predictor for employee acceptance of videoconferencing, while effort expectancy was not.

Further, Sattari et al. (2017) investigated factors that influence students’ adoption of web-based training at Tabriz University, using the UTAUT model. Two hundred twenty-nine students of Medical Science participated in this study. The result indicated that performance expectancy, effort expectancy, students’ attitude toward technology, facilitating condition, and self-efficacy positively impacted students’ intentions to adopt web-based training. Also, student anxiety negatively affected students’ intentions. However, social influence did not influence students’ behavioral intentions. Students’ behavioral intention was a significant predictor of students’ usage of web-based training.

According to the review of the literature, several published studies from various countries (Alghamdi, 2017; Escobar-Rodriguez et al., 2014; Haris & Sugito, 2015; Mahande & Malago, 2019; Ngampornchai & Adams, 2016; Tan, 2013; Uğur & Turan, 2018) have been conducted regarding the acceptance of e-learning. However, there are a few studies that focus on E-training such as Fallery et al. (2010), Sattari et al. (2017), and Zainab et al. (2017). Also, based on the literature reviewed above, most studies adopted UTAUT as the theoretical framework to examine students’ intention and use of both e-learning and E-training; therefore, UTAUT will be a suitable model as it contains the necessary studying factors for the purpose of the current study.

## Research model and hypotheses

The emergence of the UTAUT theory dates back to 2003, when Venkatesh et al. (2003) undertook a comprehensive review of eight recognized models and theories in technology acceptance, namely: the integrated model of technology acceptance and planned behavior, innovation diffusion theory (IDT), the technology acceptance model (TAM), social cognitive theory (SCT), the model of PC utilization (MPCU), theory of planned behavior (TPB), the motivational model (MM), and theory of reasoned action (TRA) (Sarabadani et al., 2017).

According to UTAUT model, four main constructs influence users’ intention towards using technology. These constructs are: Effort Expectancy (EE), Performance Expectancy (PE), Social Influence (SE), and Facilitating Conditions (FC). In addition, there are many individual factors that moderate the relationship between the aforementioned main constructs and the intention towards using the technology (Venkatesh et al., 2003). The UTAUT constructs are defined as follow:

The first construct is Performance Expectancy (PE), which is defined as the degree to which the individual thinks that using the technology will assist them in attaining job performance gains (Venkatesh et al., 2003). Previous researches stated that the relationship among UTAUT factors refers to PE having a significant effect on BI (Mahande & Malago, 2019; Sattari et al., 2017; Tan, 2013; Uğur & Turan, 2018; Venkatesh et al., 2003).

The second construct is Effort Expectancy (EE), which is defined as the degree of ease related to the use of the technology (Venkatesh et al., 2003). Previous studies indicated that EE also affected BI (Kocaleva et al., 2015; Mahande & Malago, 2019; Sattari et al., 2017; Tan, 2013; Uğur & Turan, 2018; Venkatesh et al., 2003).

The third construct is Social Influence (SI), which refers to “the degree to which an individual perceives that important others believe he or she should use the new system” (Venkatesh et al., 2003, p.451). Previous research confirmed that SI also affected BI Babie et al., 2016; Kocaleva et al., 2015; Mahande & Malago, 2019; Sattari et al., 2017; Tan, 2013; Uğur & Turan, 2018; Venkatesh et al., 2003).

The fourth construct is Facilitating Conditions (FC), which Venkatesh et al. (2003) define “as the degree to which an individual believes that an organizational and technical infrastructure exists to support use of the system” (p.453). Previous research stated that FC affected BI (Babie et al., 2016; Haris & Sugito, 2015; Kocaleva et al., 2015; Mahande & Malago, 2019; Sattari et al., 2017; Tan, 2013; Uğur & Turan, 2018; Venkatesh et al., 2003; Zainab et al., 2017).

Behavioral Intention (BI), which is a key variable in most of the accepted theories, is defined as “a person’s subjective probability that he will perform some behavior” (Fishbein & Ajzen, 1975, p. 288). Previous research states that BI affects user behavior (Haris & Sugito, 2015; Kocaleva et al., 2015; Tan, 2013; Uğur & Turan, 2018; Venkatesh et al., 2003).

Further, the present research could contribute to the existing literature in three ways:

First, prior literature has not shown enough experiential evidence to conclude the role of acceptance models such as UTAUT and its driving factors in E-training adoption (Sarabadani et al., 2017). Therefore, this study contributes to the development of the technology acceptance models as it is trying to provide empirical evidence for the impact of the UTAUT factors (i.e., PE, EE, FC, SI) on university students’ BI to adopt E-training in their field training course due to the limited number of relevant studies, especially in Saudi universities and in field training courses.

Second, applying this research in a new context (Saudi University) will increase the validity of the UTAUT framework. Furthermore, the current study tries to establish a valid and reliable scale that is suitable for the Saudi context.

Third, this research has been conducted in exceptional circumstances during the COVID-19 pandemic. These circumstances have required an immediate shifting of the regular training field course to a fully online course. No research has been conducted in such circumstances yet, therefore, the result of this study will help universities’ policymakers ensure their students’ readiness for and acceptance of E-training in such sudden crisis in the future. In addition, the factors extracted from this study could be used as guidelines for implementing E-training courses in the future.

Based on the previous studies and the aim of this current study, the researchers intended to answer two research questions:

- What are the most influencing factors that affect students’ BI toward adopting an E-training course as the result of shifting the regular field training to a fully online distance-learning course?
- Are there any significant differences between students across the acceptance factors according to their gender?

In order to answer these research questions, two hypotheses were proposed for testing:

- H1: At least one of students’ PE, EE, SI, and FC positively affect their BI toward shifting regular field training to an E-training course.
- H2: There are no significant differences between students’ BI toward shifting regular field training to an E-training course according to the students’ gender.

## The research Methodology

The main objective of the study is to investigate the most influencing factors that could affect university students’ BI toward adopting e-training as an alternative approach for delivering the regular field training course during the COVID-19 pandemic.

## Research design

In order to answer the study’s questions, quantitative and descriptive research using the deductive approach was used; survey research is commonly applied in educational studies (Cohen et al., 2011). Moreover, based on the literature, a quantitative method is the most suitable method to measure technology acceptance factors. Since this study mainly aims to statically analyze the prediction power of UTAUT factors, it uses numerical data collected via a questionnaire (Johnson & Christensen, 2008). The questionnaire items were designed mainly based on UTAUT (Venkatesh et al., 2003) and other previous studies on the acceptance of e-learning and e-training, in which a high level of validity and reliability had already been achieved. An electronic questionnaire that included 20 items based on the UTAUT model was created to gather participants’ responses. The study used a five-point Likert-type scale to allow participants to choose a number between one and five that reflected their agreement on each statement in the survey.

## Research procedure

After reviewing the literature and choosing the most suitable research method to answer the research questions, the study questionnaire was designed based on the UTAUT model. Then, the questionnaire was reviewed by experts in both education and technology fields; it was then modified and transferred to an electronic version using Google Forms. The link for the study questionnaire was sent to all registered students on the training fields’ course via email and on the course page on the Blackboard during the month of September 2020. After that, the collected data was screened and cleaned in order to deal with missing data, outliers, and invalid responses. Also, to check the reliability, Cronbach’s alpha was calculated for each item in terms of the level of internal consistency; and in addition, Exploratory Factor Analysis (EFA) was conducted using the Principal Component to evaluate the items loading in each factor.

## Research ethics

It is worth mentioning that ethical issues were taken into consideration. There were no physical risks in taking part in this study as students were only asked to participate in an electronic questionnaire, and they were informed that they were voluntarily allowed to complete the questionnaires at a convenient place and time. Furthermore, study data would be kept confidential and only used for this study's purposes.

### Population and sample

The population of the study was 429 university students aged between 20 and 26 years old. These students enrolled in the field training course in the summer semester of the academic year 2019/2020 at IAU, the participants have majored in Finance, Accounting, Administration, Management Information System, and Marketing. The data for the study were collected during the month of September 2020. The number of overall responses was 408, which represents 95% of the target study population. However, 11 responses were deleted, as they were incomplete. The statistical analysis of the research was based on 397 valid responses, which represents 92.5% of the study population.

**Fig. 1:**
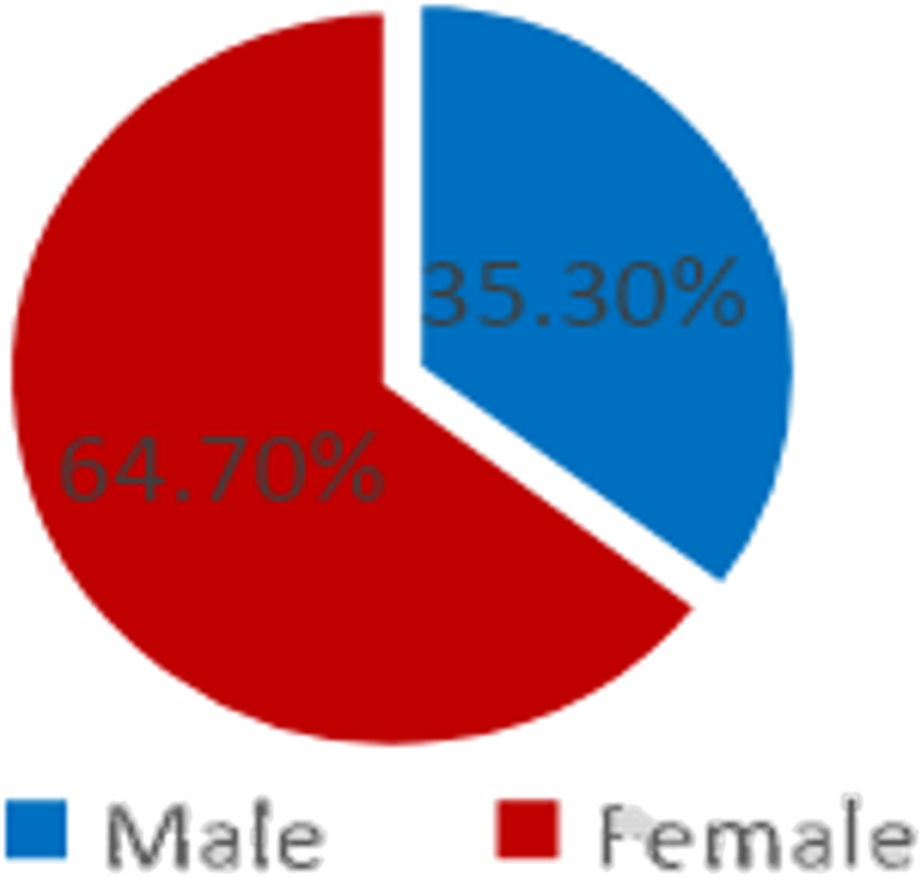
Students’ distribution according to their gender

As shown in Fig. 1, 64.7% of the sample were females, and 35.3% males, which reflects the relative distribution of gender at the university. Moreover, the participants in this study were studying different majors.

## Results and Analysis

The study, along with descriptive statistics tests, used several statistical tests, which are, EFA, Cronbach’s alpha coefficient, Correlation Coefficients, T-test, and Multiple Regression Analysis. Factor analysis allows excluding unimportant variables and retaining the more meaningful factors (Hair et al., 2010; Pallant, 2007). Therefore, the study undertook a factor analysis to examine the structure of the study model (e.g., EE, PE, SI, FC, and BI), as an advance step before applying a multiple regression test on the acceptance factors in order to answer the first research question, and a T-test to find the answer to the second research question.

A factor analysis, Principal Component Analysis, was performed. Five factors were extracted as expected when the rotation converged. As shown in Table 1, the degree of saturation of the variables of the factors is greater than 0.48; therefore, they are good components of these factors (Lo et al., 2009). Also, the extracted five factors explain 66.174% of the total variance. The KMO was 0.878, which is considered a meritorious level according to Kaiser and Rice (1974). It is a statistically significant value that the factor analysis succeeded in reducing the various factors to five, which contribute to the interpretation of the model’s Co-variance. The result of Barlett’s test for sphericity was significant (p = 0.000). Furthermore, the measure of Sampling Adequacy for all 20 items was above 0.7 (Hair et al., 2009). Therefore, it can be confirmed that the factors structure of the study is supported as they measured the scopes that they were supposed to measure.

**Table 1:** Rotated Component Matrixa

Effort Expectancy		Performance Expectancy		Facilitating condition		Behavioral intention		Social Influence	
EE1	.700	PE1	.599	FC1	.483	BI1	.629	SI1	.757
EE2	.676	PE2	.681	FC2	.809	BI2	.850	SI2	.806
EE3	.721	PE3	.808	FC3	.801	BI3	.872	SI3	.800
EE4	.742	PE4	.772						
EE5	.672	PE5	.797						
		PE6	.689						

*Extraction Method* Principal Component Analysis, *Rotation Method* Varimax with Kaiser Normalization

a. Rotation converged in 6 iterations

In order to measure the stability in the questionnaire results, Cronbach’s alpha coefficient verified the reliability of the items that result from factor analysis by analyzing the level of internal consistency for the five factors in this study. According to DeVellis (1991), a sufficient level for reliability scores should be above 0.70. As shown in Table 2, the results of each of the five factors ranged from 0.774 to 0.872, which were above a sufficient level. Moreover, the overall Cronbach’s alpha value was 0.904, which indicates very high internal reliability according to DeVellis (1991). This indicates that the study tool has great stability and validity for analyzing and interpreting the results.

**Table 2:** Results of the Reliability Analysis

Factor	N of Items	Cronbach’s Alpha
Effort Expectancy	5	.820
Performance Expectancy	6	.872
Facilitating condition	3	.774
Behavioural intention	3	.846
Social Influence	3	.814
Overall	20	.904

After confirming the factors of the study model and their items along with the level of internal consistency for the five study acceptance factors, Table 3 shows the descriptive statistics for the acceptance factors; the mean value of the five factors was between 3.72 and 4.37 out of 5, which is consider high and indicates the direction of agreement of these factors. The study also finds that the EE and the BI have the largest scores from the participants, as their response was “strongly agree”, while the other three factors (PE, FC, SI) still had a response toward “Agree” with high mean values.

**Table 3:** The descriptive statistics for the acceptance factors

Factors	Mean	Mode	Median	Std. Deviation	Direction
Effort Expectancy	4.335	4.000	4.400	0.593	Strongly Agree
Performance Expectancy	3.724	4.000	4.000	0.919	Agree
Facilitating condition	4.125	4.000	4.000	0.792	Agree
Behavioral intention	4.373	5.000	4.333	0.663	Strongly Agree
Social Influence	3.938	4.000	4.000	0.969	Agree
Valid N (listwise)	397				

For the purpose of testing the first hypothesis, the multiple regression model was used to illustrate the significant relationship between (BI) as a dependent variable, and each of the independent variables (EE-PE-FC-SI). Table 4 shows the determination coefficient and the ANOVA test for the model.

**Table 4:** Determination Coefficient and the ANOVA test for the study model

Model Summary	R	R Square		Adjusted R Square	Std. Error of the Estimate
	.572a	0.327		0.321	0.546
a. Predictors: (Constant), Social Influence, Facilitating condition, Performance Expectancy, Effort Expectancy					
ANOVA a	Sum of Squares	df	Mean Square	F	Sig
Regression	56.943	4	14.236	47.698	.000b
Residual	116.995	392	.298		
Total	173.937	396			
a. Dependent Variable: Behavioral intention					
b. Predictors: (Constant), Social Influence, Facilitating condition, Performance Expectancy, Effort Expectancy					

The value of the determining factor reached 32.1%; each of the factors (EE-PE-FC-SI) contributed by 32.1% to explain the change in the dependent variable BI. The ANOVA test demonstrates the significance of the model. The above table also indicated that the study model is statistically significant (F = 47.698, p < 0.001). Moreover, Table 5 shows the values of the regression model coefficients.

**Table 5:** Regression model coefficients

Variables	Unstandardized Coefficients		Standardized Coefficients	t	Sig
	B	Std. Error	Beta		
(Constant)	1.719	.208		8.249	.000
Effort Expectancy	.271	.059	.242	4.606	.000
Performance Expectancy	.129	.036	.178	3.534	.000
Facilitating condition	.143	.042	.171	3.388	.001
Social Influence	.104	.033	.153	3.124	.002

a. Dependent Variable: Behavioral Intention (BI)

**Fig. 2:**
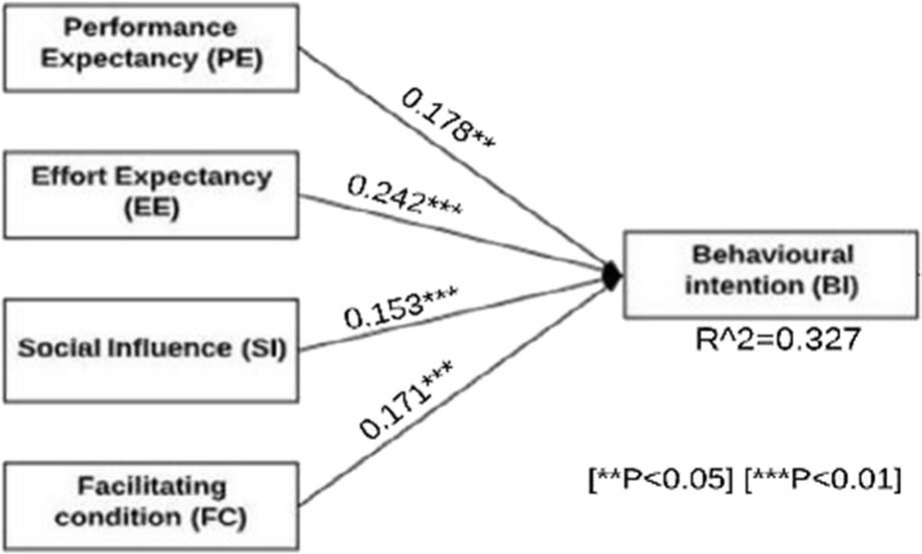
Predictive model with path coefficients

The p-values were statistically significant for each predictor (EE-PE-FC-SI), which shows that there is a positive relationship between each of these predictor factors and the dependent variable (BI). Besides, Fig. 2 shows the results of the regression models indicating that all four factors have a direct relationship with BI. It also displays the variance of the dependent variable, which was directly explained by EE, PE, FC, and SI. Therefore, this leads to accepting of the first hypothesis that there is a significant relationship between these factors. Moreover, as shown in Table 6, the correlation matrix confirms a significant relationship with a positive trend between both model-independent factors (EE-PE-FC-SI) and the dependent factor (BI). Furthermore, both FC and SI, respectively, have the highest weight in influencing students’ BI, which answers the first research question of this study regarding the most influencing factors that affect students’ BI toward adapting the shifting regular field training course to an E-training course.

**Table 6:** Correlation Coefficients matrix between model variables

Variables		Effort Expectancy	Performance Expectancy	Facilitating condition	Social Influence
Behavioral intention	Pearson Correlation	.480**	.431**	.428**	.399**
	Sig. (2-tailed)	.000	.000	.000	.000

^**^Correlation is significant at the 0.01 level (2-tailed)

Furthermore, in order to test the second hypothesis, Table 7 shows the results of the T-test, which reflect the extent of significant differences in the study factors depending on the gender of the participants. The result of the T-test indicates that there are significant differences between genders regarding three factors which are PE, FC, and SI. This supports the null hypothesis to be rejected, which leads to answering the study’s second research question regarding whether there is a significant difference between students across the acceptance factors according to their gender. However, differences between responses depending on the gender regarding BI and EE were rejected, as there were no significant differences.

**Table 7:** T-test results for significant differences in (SI) depending on gender

Independent Samples Test					
UTAUT Factors	Levene's Test for Equality of Variances		t-test for Equality of Means		
	F	Sig	t	df	Sig. (2-tailed)
Effort Expectancy	.070	.792	-.501	395	.617
			-.496	277.651	.620
Performance Expectancy	2.614	.107	2.048	395	.041
			2.091	303.640	.037
Facilitating condition	3.838	.051	2.062	395	.040
			2.200	341.788	.028
Behavioral intention	.285	.593	-.611	395	.542
			-.627	307.164	.531
Social Influence	6.108	.014	3.448	395	.001
			3.658	336.823	.000

**Fig. 3:**
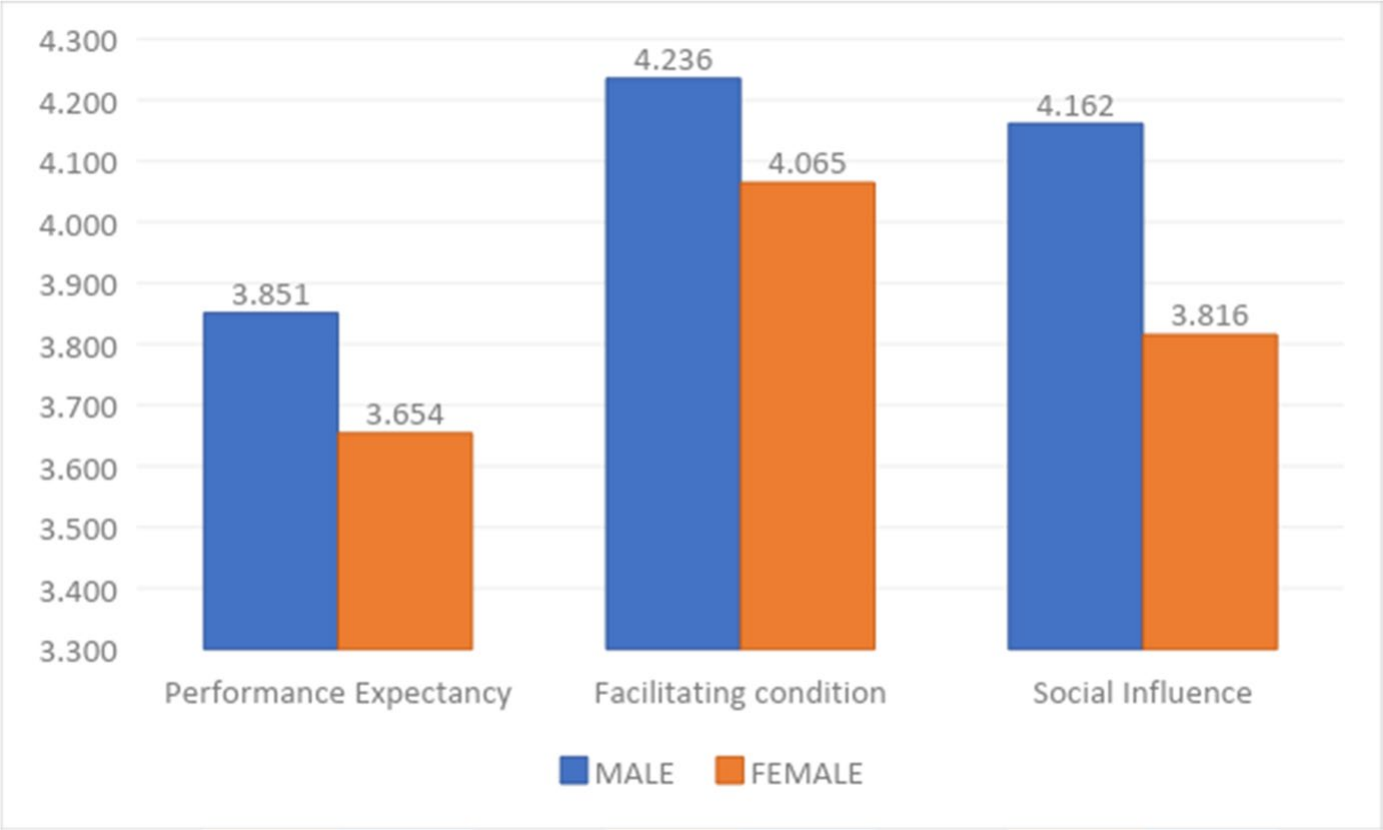
Comparing participants according to their gender for the significant factors

Regarding SI, the average for the male students reached 4.162, which is higher than the average for the female students, which was 3.816. Figure 3 shows that male students have a greater tendency toward significant differences. Similarly, the average of the male students regarding their response to FC items reached 4.236, which is higher than the average for female students, which was 4.065. Moreover, the average of the male students regarding PE reached 3.851, which is higher than the average for female students, which was 3.654.

## Discussion

This study aimed to identify the most influencing factors that affect students’ BI toward adopting learning tools to deliver field training as a distance-learning course. The results showed that all four predictor factors, which are EE, PE, FC, and SI, have a significant correlation with behavior intention. However, both FC and SI have the highest weight in influencing students’ BI toward accepting the E-training approach for delivering the field-training course. These results were mostly in line with the findings of previous studies, as will be discussed shortly, even though those studies were not directly on E-training, but on e-learning in general as only a few studies have been conducted in this regard.

Regarding the effort expectancy (EE), also known as Perceived ease of use (PEU) on the TAM model (Alghamdi, 2017), many of the prior studies (e.g., Escobar-Rodriguez et al., 2014; Mahande & Malago, 2019; Ngampornchai & Adams, 2016; Tan, 2013; Tarhini et al., 2015; Uğur & Turan, 2018; Vululleh, 2018) which conducted their researches on e-learning acceptance along with Sattari et al. (2017) and Zainab et al. (2017) who conducted their studies on E-training acceptance, found that there is a statically significant relationship between PE and BI. However, both Alghamdi (2017), who conducted his research on university students, and Fallery et al. (2010), who conducted their study on employees, reported no significant direct relation from PE to BI. The result was not in line with the current study either because these studies were conducted before the technology revolution took place in the last few years and due to the simple type of study as in Fallery et al. (2010) or because of the different aim of the study as in Alghamdi (2017) who was looking for the acceptance of using mobile devices as e-learning tools. It is important to note that EE in the current study shows the highest impact on students’ BI regarding adopting E-training for field training courses.

Furthermore, the current study confirms that significant Performance Expectancy (PE), which can also be named Perceived usefulness (PU) as in the TAM model and other theories, has a statistically significant effect on BI. This finding is totally in line with the results of many of the previous studies on e-learning acceptance (e.g., Alghamdi, 2017; Escobar-Rodriguez et al., 2014; Mahande & Malago, 2019; Ngampornchai & Adams, 2016; Tan, 2013; Tarhini et al., 2015; Uğur & Turan, 2018; Venkatesh et al., 2003; Vululleh, 2018), and also in line with previous studies on E-training acceptance (e.g., Fallery et al., 2010; Sattari et al., 2017; Zainab et al., 2017). The finding denoted that the relationship between PE and BI is strong (Venkatesh et al., 2003), which implies considering these two factors before adopting E-training for field training course.

Regarding the effect of Social influence (SI) on students’ BI and the relation between them, the current study indicated that there is a relation between the two factors and the impact of SI on students’ BI, which confirms the relation between those factors as in the UTAUT model (Venkatesh et al., 2003). This finding was in line with several prior studies on e-learning acceptance among university students (Alghamdi, 2017; Escobar-Rodriguez et al., 2014; Ngampornchai & Adams, 2016; Tan, 2013). In contrast to the current study finding, both Sattari et al. (2017) and Mahande and Malago (2019) reported no relation in their studies between SI and BI, which is not common in relevant studies and the fundamental model of UTAUT (Venkatesh et al., 2003) and extended model of UTAUT or the extended model of UTAUT (Venkatesh et al., 2012).

Furthermore, the current study also confirms that there is an effect on students’ BI from the Facilitating conditions (FC) factors, as indicated in the UTAUT module (Venkatesh et al., 2003). Most previous studies confirm similar findings (e.g., Alghamdi, 2017; Mahande & Malago, 2019; Sattari et al., 2017; Tan, 2013; Tarhini et al., 2015). This shows the importance of the FC regarding adopting technology such as E-training tools in the current study.

Moreover, it was investigated whether there are any significant differences between students regarding their gender across the acceptance factors. The finding of the current study indicated that there are significant differences between genders regarding three factors, which are PE, FC, and SI. Moreover, the results show that male students have a higher average score on those three factors than female students. In relation to previous studies, no study that the authors of this study are aware of during the time of writing this paper has reviewed the differences between male and female university students regarding their opinion on the acceptance factors related to adopting an E-training approach alternative to the regular field training course. However, Alghamdi (2017) who conducted his study on the acceptance of smart mobile devices as learning tools for university students in three universities in Saudi Arabia, found that female students had higher mean scores across PU, which is equivalent to PE and BI.

Nevertheless, both males and females had an identical mean average score on PEU, equal to EE in the current study. Alghamdi (2017) also reported there were significant differences between students attributed to their gender according to the study acceptance factors. Alghamdi’s findings were in line with the current study regarding the significant differences between students regarding their gender across PE and BI.

## Conclusion

Given that the COVID-19 pandemic precluded many universities and colleges worldwide from continuing to offer their courses in the face-to-face format, all courses in the IAU like other universities worldwide were delivered via distance learning, including the field-training course. Since this was the first time that the field-training course had been offered in a distance format, the present study contributes to a better understanding of university students’ intention to accept e-training. This study examined the predicted factors that have most influenced students’ acceptance of e-training. By using an electronic questionnaire, the study data were collected from 397 university students who enrolled in the field training course and had majored in Finance, Accounting, Administration, Management Information System, and Marketing. The result revealed that PE, PU, FC were significant predictors for students’ acceptance of e-training.

As far as the implications of this study are concerned, the study results could assist the IAU decision-makers to determine to what extent IAU students accept e-training. Also, this result could be generalized to other universities in the region. In addition, this study provides university decision-makers a clear image of the most significant factors that motivate or hinder students' acceptance of e-training. This study found that PE, PU, FC were the main factors in predicting student acceptance of e-training. As result, in providing workshops and professional development on how to develop and deliver an e-training course for academic departments and colleges, offering training for students to improve their technical skills that might be required in such courses, increasing faculty members, students, and a university community awareness about the benefits of accepting e-training, as well as providing the required technology infrastructure along with technical support teams, it would be important to target PE, PU, FC and this, in turn, will positively affect students’ intention to accept e-training as an approach for delivering field-training courses.
